# Ascorbic Acid Treatments as Effective and Safe Anti-Aging Therapies for Sensitive Skin

**DOI:** 10.3390/antiox13020174

**Published:** 2024-01-30

**Authors:** Anna Jaros-Sajda, Elzbieta Budzisz, Anna Erkiert-Polguj

**Affiliations:** 1 Department of Cosmetic Raw Materials Chemistry, Faculty of Pharmacy, Medical University of Lodz, 90-419 Lodz, Polandelzbieta.budzisz@umed.lodz.pl (E.B.); 2 Department of Cosmetology and Aesthetic Dermatology, Faculty of Pharmacy, Medical University of Lodz, 90-419 Lodz, Poland

**Keywords:** sensitive skin, ascorbic acid, microneedling

## Abstract

The most common signs of aging skin include a decrease in firmness and density, uneven skin tone, and a tendency to erythema. There is an ever-increasing interest in aesthetic treatments that maintain the skin’s favorable appearance. However, such therapies are difficult in the case of sensitive skin, defined as a set of stimuli-triggered symptoms (stinging, erythema, burning, and itching) that would not appear in healthy skin. Sensitive skin is common and affects, to varying degrees, about half of the European population. This study was aimed at evaluating the effects of ascorbic acid—a known antioxidant—applied with sonophoresis and microneedling on the signs of photoaging in reactive and erythematous skin. A significant improvement in skin elasticity was observed after a series of tests. A significant reduction in erythema was observed after both therapies. The greatest reduction was observed on the cheeks after applying vitamin C combined with microneedling. At the same time, the results showed an excellent tolerance of both treatments, which proved them to be safe and effective.

## 1. Introduction

Skin condition is influenced not only by the passage of time but also by the effects of various endogenous and exogenous factors [1]. The amount of elastic fibers decreases whilst their fragmentation increases; collagen fibers become thinner and more distant from each other; and their arrangement is reorganized [2,3]. Furthermore, their production by fibroblasts decreases, while, at the same time, the activity of metalloproteinases (MMPs), which break down collagen, increases. Atrophy of the stratum granulosum and the spinous layer results in the thinning of the epidermis and thus leads to greater visibility of the blood vessels. The functions of the sebaceous gland weaken with age. This, in turn, contributes to greater water loss, as well as abnormalities in the protective hydrolipid coat of the skin. The pigmentation associated with aging can include changes in the number of melanocytes, leading to hyperpigmentation or hypopigmentation. Wrinkles, discolorations, changes in the facial oval, and other cosmetic defects that appear with age result in reduced self-esteem, and deterioration in the quality of daily life and social relations. For this reason, there has been a steady increase in interest in aesthetic skin treatments aimed at rejuvenating the skin, maintaining its favorable appearance, and slowing down the aging process [4].

Such therapies are more difficult in the case of sensitive skin, which is defined as a set of unpleasant symptoms (stinging, erythema, burning, and itching), resulting from stimuli that should not cause any reactions in healthy skin [5]. Sensitive skin constitutes a common problem that affects, to varying degrees, about half of the European population and is most common in women [6]. The pathophysiology of sensitive skin is not yet fully understood, although its pathomechanism is thought to be related to overactivity of the cutaneous nervous system, particularly the activation of sensory proteins located on the keranocytes and nerve endings [6,7] and damage to the lipid barrier [8]. Most often, the symptoms of sensitive skin are aggravated by external factors such as UV radiation, wind, temperature extremes, spicy foods, and chemicals [9]. The diagnosis of sensitive skin is complex, and the literature still does not offer a clear definition based on specific parameters. Nevertheless, some of the literature relies on the qualification of sensitive skin with the use of questionnaires with probands and assessing visible dryness and erythema [6,10].

Furthermore, in the process of aging and in sensitive skin, the role of reactive oxygen species (ROS) should be taken into account. The skin, as the external part of the body, is constantly exposed to ultraviolet (UV) radiation, which leads to the production of ROS. Antioxidants are needed in the stratum corneum to neutralize the ROS, and they consist of lipophylic and hydrophylic low molecular weight antioxidants (e.g., carotenoids), vitamins, enzymes, and other antioxidants (e.g., melanin and coenzyme Q). Unfortunately, with age and stress, antioxidants are depleted, and, in consequence, the skin loses the protective system. The replacement of antioxidants can be conducted in oral and topical ways [11].

One of the active substances used in the photoaging treatments is ascorbic acid (vitamin C). It not only stimulates collagen synthesis but also acts as a cofactor for collagen-stabilizing lysine and proline hydroxylases and inhibits metalloproteinase-1 (MMP-1) activity [12]. It also provides protection from elastin damage and inhibits the cross-linking effect that is formed in wrinkles [13]. Vitamin C also inhibits melanogenesis, and, as a result, it can be used in reducing skin hyperpigmentation. Ascorbic acid indicates antioxidant properties and protects skin from UV radiation by neutralizing free radicals [14].

The biodisposability of most topical formulations is low, and the ineffective delivery of active substances into the deeper epidermis layers or into the dermis may be a problem with a lack of or poor results for aesthetic therapies. Needle mesotherapy can be used to deliver substances into the dermis, but the pain and use of medical needles can be a barrier for some people. Furthermore, taking into consideration erythematous and sensitive skin, we have to remember that irritation after needle mesotherapy can be greater and last longer, which can also not be acceptable. Although ascorbic acid is a small protein of 176 Da, the epidermis is a great barrier, and, in order to facilitate the penetration of active substances through the stratum corneum, this study used microneedle mesotherapy and sonophoresis. Microneedle mesotherapy is a method that aims at stimulating the regeneration of collagen and elastin fibers through the use of mechanical stimulation—micropunctures combined with the introduction of the active substance into the deeper layers of the epidermis. Because we decided to compare the effects of ascorbic acid, we have chosen 0.2 mm of needles to obtain only the effect of a better penetration of ascorbic acid, not to start a real skin regeneration after longer needles microneedling [15,16]. Sonophoresis was the second method used during this study. During sonophoresis, ultrasonic waves improve the transfer of active substances deep into the skin by increasing cell permeability and the kinetic energy of the active substance particles. Ultrasounds of frequency 2 MHz was used. During high frequency ultrasound, cavitation bubbles seem to have the most important effect. These bubbles can interact and modify the skin barrier by inducing, dilating, and connecting defects to form regions of increased permeability within the stratum corneum [17].

The aim of this study was to evaluate the effects of ascorbic acid applied using sonophoresis and microneedling on the signs of photoaging in people with reactive and erythematous skin.

## 2. Materials and Methods

This study included twenty-five healthy probands within the age range of 25–63 years with noticeable erythematous changes and sensitive skin. The criterion for qualifying a proband’s skin as sensitive was based on initial questionnaires indicating the factors causing increased erythema and skin discomfort (UV exposure, high temperatures, exercise, alcohol intake, and spicy food). Initial evaluations of erythema, TEWL, and hydration were used as additional criteria as well. The exclusion criteria included pregnancy and lactation, active viral lesions on the facial skin, active fungal and bacterial diseases, and participation in other dermatological therapies during the study period and within the previous 6 months. An injectable solution containing 500 mg of ascorbic acid (100 mg/mL; Teva, Warsaw, Poland) was applied to the facial skin with microneedle mesotherapy (0.2 mm) (on the right side of the face) or sonophoresis (on the left side of the face). The therapy consisted of six series of treatments performed at two-week intervals. The microneedle mesotherapy was applied on the right side of the face using a sterile tip. The microneedle equipment (Dr. Pen, China), was applied at 0.2 mm for 3–5 min to the slight erythema. On the left part of the face, sonication was carried out in the continuous mode, at an intensity of 1–1.5 W/cm^2^ and a frequency of 2 MHz for 5 min (SY089, Zhong Shan Syou EiBeauty Instrument, Zhongshan, Guangdong, China).

**Figure 1:**
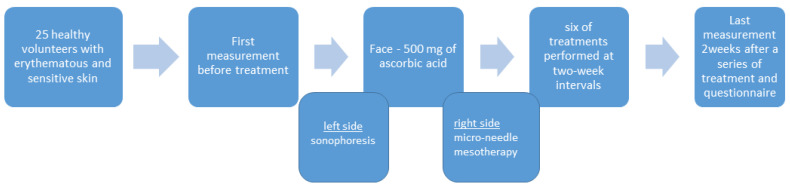
A summary of the research protocol.

The skin condition was assessed before the treatment and two weeks after the last, sixth session. The severity of the erythema and pigmentation, as well as hydration and changes in transepidermal water loss (TEWL), were measured with an MPA 580 (Courage & Khazaka Electronic GmbH, Cologne, Germany). Skin parameters, such as the skin’s ability to return to baseline (R1) and net elasticity (R5), were assessed with a Cutometer^®^ device (Courage-Khazaka, Cologne, Germany). Its measuring principle is based on the suction method, by which negative pressure deforms the skin mechanically. Subsequent measurement of the parameters indicate the skin’s ability to return to its initial state. The measurements were carried out in the same room, under constant conditions (a temperature of 20 °C and a humidity of 45% ± 5%), once the participants had been acclimatized to ambient conditions for 15 min. Besides the skin parameter measurements, the patients were also asked fill out a questionnaire in which they evaluated the treatment effects that they observed (Figure 1).

This study was conducted in accordance with the guidelines of the Declaration of Helsinki of 1964, as amended, and was approved by the Medical University bioethics committee. All women participating in this study signed an informed consent form.

### Statistical Analysis

The mean and standard deviation (mean ± SD) were used to describe the normal distribution parameters. The differences in the skin parameter percentage changes from baseline values were calculated with the following formula: ((([x(t_1_) − x(t_0_)]/x(t_0_)) × 100). The variables that did not have a normal distribution were expressed as the median and quartile range (median (25%; 75%)). In order to evaluate the differences in skin parameters over time, the *t*-test was used for the data with a normal distribution, while the Wilcoxon rank test was used for data that deviated from a normal distribution. The Mann–Whitney test was used to statistically evaluate the differences between the independent samples with distributions deviating from normal. A statistical evaluation of the categorical variables was performed using either chi-square tests or Fisher’s exact test. The *p* values lower than 0.05 were considered statistically significant.

## 3. Results

Based on the initial assessment, the skin of all probands was classified as sensitive, with varying degrees of severity. The probands indicated that their skin reacted with increased redness to irritants such as alcohol, spicy foods, UV exposure, stress, large temperature amplitudes, medications, physical exercise, and the sauna. In addition, the tests performed with the MPA 580 (Courage & Khazaka Electronic GmbH, Cologne, Germany) showed that all probands from the Mexametr probe test group had an initial score > 300, which classifies the skin as erythematous according to the manufacturer’s interpretation of the results. In addition, the hydration measurements showed that 34% of the probands suffered from dry skin.

**Figure 2:**
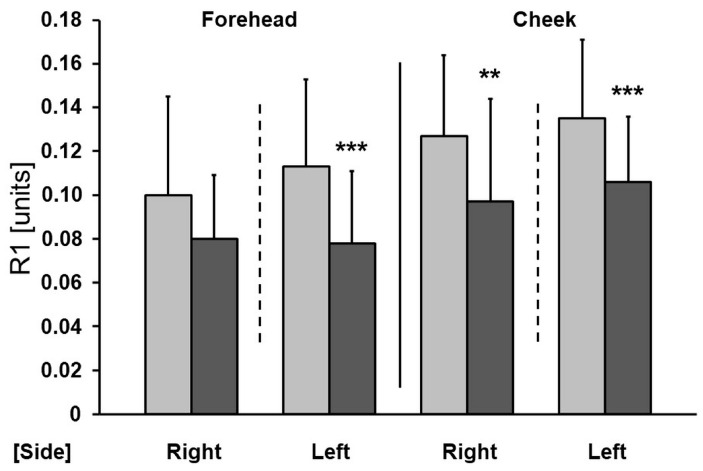
Changes in the R1 parameter as a result of the therapy (right—microneedle mesotherapy; left—sonophoresis). Grey—before treatment, black—after treatment. ** *p* < 0.01, *** *p* < 0.001.

**Figure 3:**
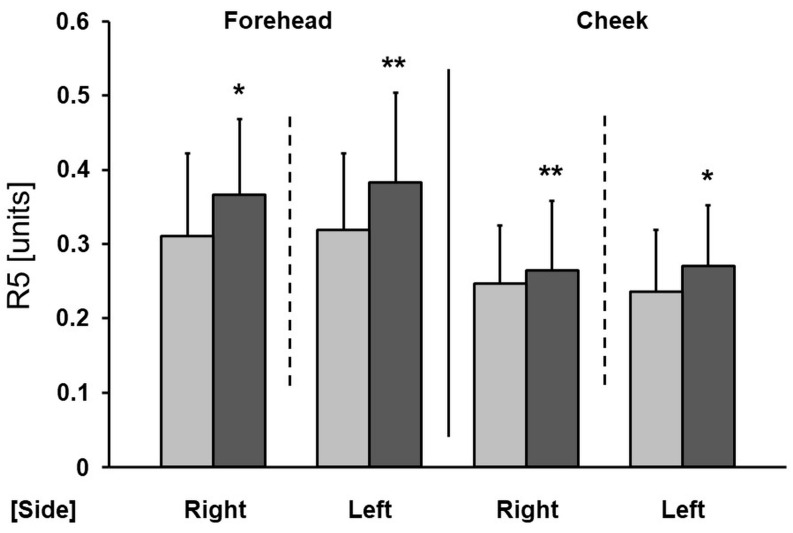
Changes in the R5 parameter as a result of the therapy (right—microneedle mesotherapy; left—sonophoresis). Grey—before treatment, black—after treatment. * *p* < 0.05, ** *p* < 0.01.

In this study we observed a significant improvement in skin elasticity. After a series of tests, there was a statistically significant decrease in the R1 parameter on the forehead and cheeks (except for the forehead on the left side) (Figure 2) and a significant increase in the R5 parameter on the forehead and cheeks (except for the cheek on the right side) (Figure 3). The greatest reduction in the R1 parameter was found on both cheeks (a decrease of 25.3% on the left side and 28.2% on the right side).

**Figure 4:**
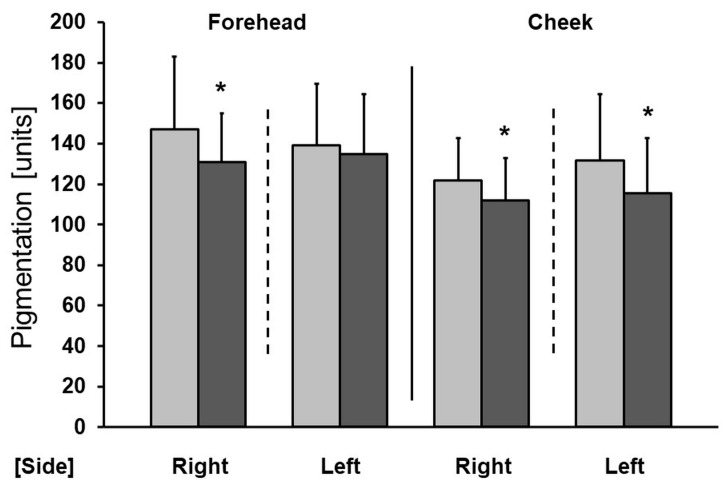
Improvement in pigmentation after a series of treatments (right—microneedle mesotherapy; left—sonophoresis). Grey—before treatment, black—after treatment. * *p* < 0.05.

Both therapies resulted in a significant reduction in pigmentation on the cheeks, while a significant improvement on the forehead was noted only after a series of microneedle mesotherapy treatments (Figure 4).

**Figure 5:**
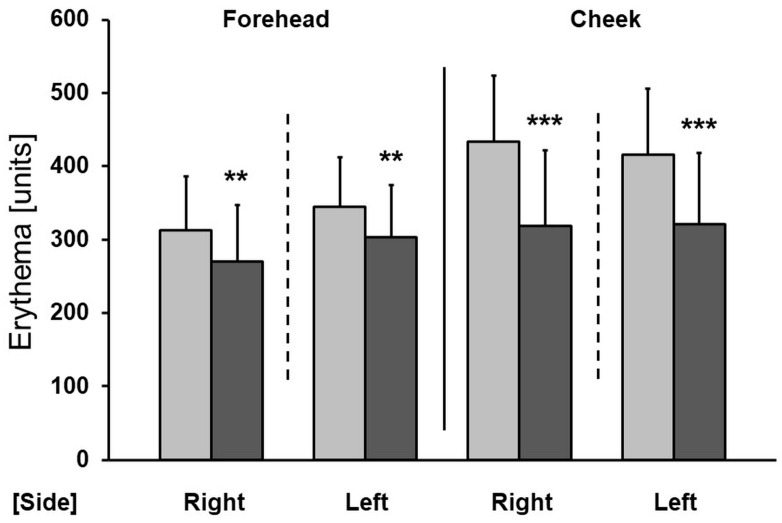
Improvement in erythema after a series of treatments (right—microneedle mesotherapy; left—sonophoresis). Grey—before treatment, black—after treatment. ** *p* < 0.01, *** *p* < 0.001.

A significant reduction in erythema was also observed after both therapies, both on the cheeks and forehead (Figure 5). The greatest reduction was observed on the cheeks, following the application of vitamin C combined with microneedle mesotherapy (a reduction of 23.5%). Sonophoresis + ascorbic acid resulted in a 19.3% reduction in erythema on the cheeks. On the forehead, the improvement amounted to 8.8%.

In the group of participants with dehydrated skin, it was noted that the use of ascorbic acid and sonophoresis improved the degree of hydration in eight probands, while the combination of ascorbic acid and microneedle mesotherapy showed improvements in nine probands, changing the skin from dry to adequately hydrated.

Furthermore, the use of ascorbic acid in combination with both microneedle mesotherapy and sonophoresis reduced transepidermal water loss. However, the results were not statistically significant, due to the fact that part of the proband group already showed initial readings that were deemed to be characteristic of healthy skin with a proper hydrolipid coat.

The treatment effects were also confirmed by photo analysis.

Upon completion of the treatment series, the participants were asked to fill out a questionnaire in which they evaluated the treatment effects that they had observed.

**Figure 6:**
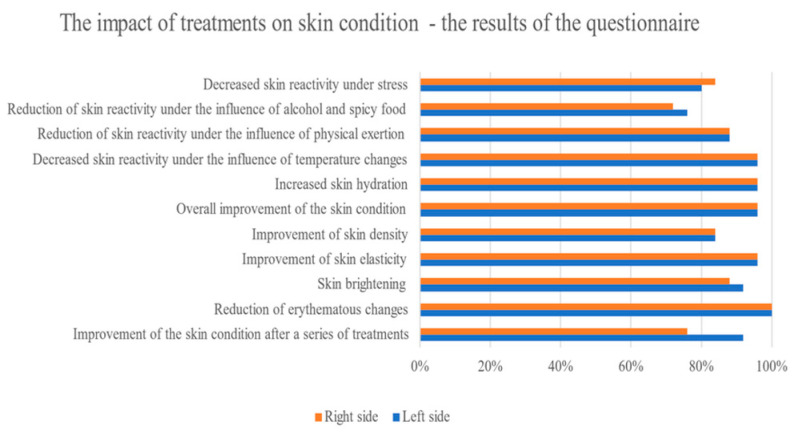
Subjective perception of changes in skin condition after a series of treatments.

The questionnaire results showed that all participants noted a reduction in erythematous lesions, and 96% of them reported an overall improvement in skin condition, increased elasticity, and increased hydration. What seems particularly important is the reduction in skin reactivity to external factors such as temperature, alcohol and spicy foods, physical exertion, and stress, as noted by the probands. (Figure 6). There were 92% of probands who noticed a brightening of the skin on the side of the face where sonophoresis was applied (compared to 88% after the microneedle mesotherapy). Both therapies comparably improved the skin density according to the participants (in 84% of the probands). The participants noticed the greatest improvement in the skin condition after the fourth treatment, while the very first improvements in the skin condition appeared after just 2–3 treatments (Figure 6).

At the same time, the results showed a very good tolerance of the treatments. Only three participants reported skin irritation on the left side, while four participants reported skin irritation on the right side, but this effect disappeared a few hours after each treatment.

**Figure 7:**
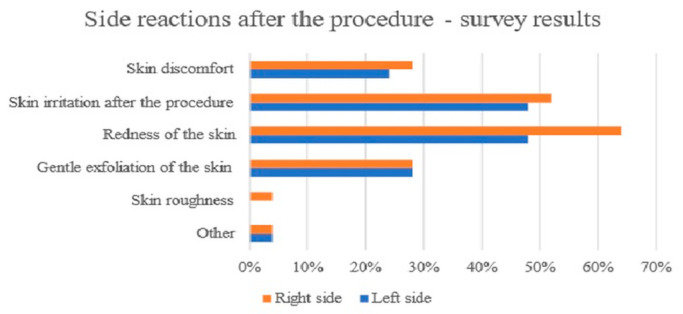
Side reactions after the procedure.

Fewer than 30% of participants experienced discomfort following the treatments. Side reactions in the form of skin irritation or erythema were observed immediately after the therapy by half of participants (48% after sonophoresis, 52% after microneedle mesotherapy). However, in the majority of women, these reactions disappeared within several minutes; in a minority, the effect lasted for several hours. Eight participants reported mild peeling of the skin after the treatment, and one reported skin roughness (Figure 7).

## 4. Discussion

Sensitive skin is nowadays clinically defined as the appearance of the abnormal sensations of burning, itching, and stinging, as well as erythematous symptoms, in response to a variety of physical (high or low temperature, wind, and UV radiation), chemical (cosmetics, soaps, and water), and sometimes psychological (stress) factors [18,19]. The pathophysiology of sensitive skin is complex. Among other things, researchers link the problem to the excessive involvement of the cutaneous nervous system. Neurotransmitters, such as calcitonin gene-related peptide (CGRP), Substance *p*, and vasoactive intestinal peptide (VIP), determine the development of neurogenic inflammation with vasodilation and mast cell degranulation [20]. Special attention is also paid to the role of TRP channels, which are the only molecules that can be activated by both physical and chemical factors. Despite the lack of conclusive studies, their either abnormal or increased activity is indicated in cases of sensitive skin. The TRP channels are expressed on nerve endings, keratinocytes, and Merkel cells [18,21]. For example, TRPV1 is activated by capsaicin, heat, and H+ ions, and TRPV4 is activated by heat and mechanical stress. The TRP channels are also activated by substances found in cosmetics [22]. The role of the hydrolipidic coat is emphasized in sensitive skin as well. A damaged barrier leads to TEWL, which in turn contributes to contact with precipitating agents [23].

Sensitive skin is also often referred to as reactive skin, intolerant skin, or irritable skin [18], which underlines what a therapeutic challenge it is to deliver effective and safe anti-aging therapies to patients with this type of skin. Nevertheless, we achieved very good therapeutic results in this study, because improvements in skin elasticity, the reduction in erythema, the reduction in pigmentation, and the restoration of adequate hydration were noted. At the same time, there were no long-term adverse reactions or a stronger skin sensitization. It was only immediately after the treatment, depending on the application method used, that the probands indicated the occurrence of redness (64% after microneedle mesotherapy, 48% after sonophoresis) and a general feeling of skin irritation (48% after sonophoresis, 52% after microneedle mesotherapy). However, in most women, these reactions disappeared within several minutes; in a minority, the effect lasted for several hours.

A significant improvement in skin elasticity, reflected in a statistically significant decrease in the R1 parameter and a significant increase in the R5 parameter, was observed. A decrease in R1 indicates an improvement in skin condition, as does the increase in R5. Similar conclusions were drawn from a randomized clinical trial, where increased mRNA levels of type I and type III collagen were confirmed in a skin biopsy of women using a cream containing 5% ascorbic acid [24]. Another study also showed that topically applied vitamin C can protect the skin from UV damage by reducing the formation of free radicals [25]. By acting as an antioxidant, it also removes reactive oxygen species or free radicals [26]. In a randomized, double-blind, placebo-controlled study involving healthy women, the topical application of a 5% ascorbic acid cream (once a day for a period of 6 months) to sun-exposed parts of the body significantly reduced deep wrinkles and improved skin texture compared to treatment with an excipient [27]. Furthermore, in a double-blind, placebo-controlled clinical trial, the use of a formulation containing 10% ascorbic acid for a period of 12 weeks significantly reduced signs of photoaging and reduced wrinkles compared to a placebo [25]. Anti-aging properties ware also confirmed with the use of 3% ascorbic acid for 4 months in a clinical trial, where it significantly increased the density of the papillary layer of skin [28]. Escobar et al. [29] also included in their anti-aging study persons with sensitive skin. The use of a cosmetic with 10% vitamin C, biopeptides, and hyaluronic acid led to wrinkle shallowing. It seems that the aforementioned anti-aging effect of ascorbic acid is based on both the antioxidant properties of vitamin C and its role as a cofactor in proper collagen production. Vitamin C also impairs the expression of metalloproteinases 1 and 2 after the UVA induction [30]. The metalloproteinases are overexpressed in aging skin, leading to collagen and other parts of extracellular matrix degeneration [31]. The impact of vitamin C on metalloproteinases is due to its antioxidant potency.

Aging skin is also characterized by abnormal pigmentation and the loss of hydration. In this study, vitamin C improved the appearance of skin in this aspect as well. Both sonophoresis and microneedle mesotherapy led to a significant reduction in pigmentation on the cheeks, while microneedle mesotherapy also produced a beneficial effect on the forehead. Ascorbic acid is an important depigmenting agent, because it inhibits tyrosinase, thereby reducing the formation of melanin [32]. The application of a formulation containing 25% vitamin C and a chemical compound that improves its penetration for a period of 16 weeks resulted in a significant reduction in melasma-induced pigmentation [33]. Furthermore, the results of another study conducted on 35 women over the age of 40 show that the day-to-day (once a day for 56 days) application of a serum containing vitamins C and E combined with palmitoyl tripeptide-38 resulted in a visible improvement in skin appearance (smoothing and brightening) and texture [34].

The use of ascorbic acid had a beneficial effect on hydration by restoring normal parameters on the cheeks in 32% of the probands undergoing sonophoresis and 36% of the probands undergoing microneedle mesotherapy. In addition, ascorbic acid combined with microneedle mesotherapy indicated a significant statistical reduction in transepidermal water loss on the cheeks. In another study conducted on cell lines, the application of vitamin C was shown to improve the lipid barrier and stimulate the differentiation of keratinocytes, and thus may contribute to the thickening of the stratum corneum, which ensures the integrity of the skin barrier and results in improved water retention [14,35]. A strengthened hydrolipid barrier reduces contact with precipitating factors [18], thus making the applied therapy beneficial for sensitive skins.

In addition to reactivity to external factors, sensitive skin is often accompanied by erythema [18,19]. The appearance of facial erythema (both episodical and permanent) is often associated with sun exposure. UV radiation leads to the formation of a large number of free radicals and, consequently, oxidative stress, so the antioxidant properties of vitamin C become very important [36]. In this study, a significant reduction in erythema was obtained with both sonophoresis and microneedle mesotherapy. The best effects were found on the cheeks after applying vitamin C combined with microneedle mesotherapy (a 23.5% improvement). The reduction in erythema on the forehead was lower, which may be due to the fact that superficial dilated blood vessels are most often seen on the cheeks, where the skin is thinner and more delicate than in other parts of the face, and the baseline measurements were significantly higher there. One animal study showed that the topical application of 10% vitamin C reduced UVB-induced erythema by 52% [37]. Preparations containing vitamin C have also been studied for reducing erythema in rosacea, which in most cases is accompanied by sensitive skin [38]. The daily application of a cosmetic preparation of 5.0% L-ascorbic acid was shown to produce both an objective and a subjective improvement in erythema [38]. The authors suggested that the production of free radicals may play a role in the inflammatory response of rosacea, and the antioxidant effect of L-ascorbic acid may be responsible for the effectiveness of the therapy. It appears that the aforementioned increase in the epidermal thickness may also be associated with a reduced appearance of dilated blood vessels, resulting in reduced redness. Ascorbic acid seals blood vessels and makes them less fragile, which constitutes another mechanism by which it effectively combats erythema and telangiectasias.

The above effects were confirmed by participants who observed an overall improvement in skin condition; reduced erythema; an increase in elasticity, density, and hydration; and a brightening of the skin. The probands also indicated a significant reduction in skin reactivity due to alcohol and spicy foods; physical exercise; temperature changes; and stress.

## 5. Conclusions

In conclusion, the results of this study, along with other clinical studies, confirm that the application of ascorbic acid contributes to wrinkle reduction and increased skin elasticity by preventing collagen loss through photoaging and natural skin aging [39]. The anti-aging effects observed in this study are not solely due to the use of pure ascorbic acid but also microneedle mesotherapy and sonophoresis. This study showed that the therapy works very well on sensitive skin. An important effect of ascorbic acid is the reduction in erythema. Sensitive, reactive, erythema-prone skin is a particular challenge for dermatologists. The formulation and the two application techniques used in this study significantly alleviated redness and reduced skin reactivity.

This study also showed that the therapies used proved to be effective and safe, as they caused only mild skin irritation in some of the study participants, which disappeared after a few minutes to a few hours after the therapy.

## Data Availability

Data available on request.
